# Development of a prognostic model incorporating a cuproptosis-related signature and CNN3 as a predictor in childhood acute myelocytic leukemia

**DOI:** 10.3389/fonc.2024.1494777

**Published:** 2024-11-01

**Authors:** Jiafan Cao, Mengyun Xie, Kexin Sun, Yijun Zhao, Jiayin Zheng, Ying Wang, Yucan Zheng, Sixi Liu, Uet Yu

**Affiliations:** Department of Hematology and Oncology, Shenzhen Children’s Hospital, Shenzhen, China

**Keywords:** acute myelocytic leukemia, cuproptosis, CNN3, prognostic model, immune cell phenotypes

## Abstract

**Background:**

Childhood acute myeloid leukemia (cAML) is the second most common pediatric blood cancer, with high heterogeneity and poor prognosis. Recent studies have highlighted cuproptosis, a newly discovered form of programmed cell death triggered by the accumulation of intracellular copper ions, as a critical mechanism influencing cancer survival and resistance. Given its emerging role in cancer biology, we investigated cuproptosis-related genes (CRGs) in cAML to explore their potential in prognostic prediction and therapeutic targeting.

**Methods:**

Gene expression data from publicly available sources were analyzed to identify differentially expressed CRGs. Samples were categorized based on their expression profiles, followed by the development of a prognostic risk model using multivariable Cox regression, LASSO, and univariable analyses. The model’s performance was evaluated through Kaplan-Meier survival analysis and ROC analysis. Immune infiltration in the tumor microenvironment was assessed using ssGSEA, validated by CIBERSORT. Drug sensitivity correlations were analyzed, and functional validation experiments were conducted on THP-1 and MOLM13 cell lines to assess the role of *CNN3*.

**Results:**

A set of 12 differential CRGs was used to build a robust prognostic risk model, with high accuracy in predicting patient outcomes (P < 0.001). Significant differences in immune cell composition were identified between risk groups, particularly in T cells, B cells, monocytes, and dendritic cells. Drug sensitivity analysis revealed altered IC50 values for drugs like 5-fluorouracil and bortezomib. Knockdown of CNN3 in leukemia cell lines led to reduced cell proliferation.

**Conclusion:**

Our CRGs-based prognostic model shows potential for guiding personalized treatment strategies in cAML. The differences in immune cell infiltration between risk groups suggest that immune modulation is key in cAML progression. *CNN3* and *LGR4* were identified as modulators of cAML progression, making them potential therapeutic targets. Future studies with larger cohorts are essential to validate these findings and further explore CRGs-targeted therapies.

## 1 Introduction

Acute leukemia represents the most prevalent type of cancer in children, with its incidence on the rise (1). Although acute myeloid leukemia (AML) accounts for only 20% of all pediatric acute leukemia cases, it has surpassed acute lymphoblastic leukemia (ALL) as the primary cause of leukemia-related mortality among children. A significant factor contributing to this shift is the failure of current prognostic models, which often misclassify children who eventually succumb to the disease as being at low or intermediate risk. Moreover, effective targeted therapies for childhood AML (cAML) remain scarce, with investigational tyrosine kinase inhibitors for FLT3-activated AML being among the few under exploration. This scarcity contrasts sharply with the treatment landscape for adult AML, where actionable mutations like IDH play a key role in guiding therapy. In cAML, the absence of such common mutations reflects the disease’s distinct molecular makeup, complicating the development of treatment options (2, 3). These challenges, including the lack of effective targeted therapies and the limitations of current prognostic models, stem largely from an incomplete understanding of cAML’s underlying biology (4).

Cuproptosis, or copper-induced cell death, is a recently discovered form of cell death linked to mitochondrial metabolism, distinct from well-known mechanisms like apoptosis, ferroptosis, and necroptosis (5). Studies indicate that mitochondrial respiration plays a pivotal role in cuproptosis, which is triggered by either a deficiency or excess of intracellular copper. A lack of copper impairs the activity of copper-binding enzymes (6). In contrast, when copper levels are elevated, it can accumulate in the mitochondria and bind to lipid-acylated components of the tricarboxylic acid (TCA) cycle. This interaction induces toxic protein stress, ultimately resulting in cell death (7). Research on muscle-invasive bladder cancer has identified a cuproptosis-related genes (CRGs) signature with implications for prognosis and treatment (8). In adult AML, cuproptosis-related lncRNA have been linked to patient prognosis and immune characteristics, but its role in cAML remains unexplored (9).

Given this context, our research delved into how cuproptosis intersects with cAML, specifically investigating its impact on the tumor microenvironment, potential drug resistance, and interactions with immune cells. We aimed to create a reliable model for predicting cAML outcomes by integrating these factors. To ensure the accuracy of our predictions, we not only developed this prognostic model but also carried out experimental validations *in vitro* to confirm the role of the genes incorporated into our model. This dual approach—combining computational predictions with experimental validation—helped us better understand the complex dynamics of cuproptosis in cAML and its implications for treatment strategies.

## 2 Materials and methods

### 2.1 Dataset information

We gathered mRNA sequencing data from 187 pediatric cAML patients through the TARGET (Therapeutically Applicable Research to Generate Effective Treatments) program, which collaborates with The Cancer Genome Atlas (TCGA). TARGET is a database focused on pediatric cancer research, aimed at improving treatment outcomes by providing comprehensive genomic, transcriptomic, and clinical data. The database compiles a wealth of samples from various types of childhood cancers, helping researchers identify potential therapeutic targets and biomarkers for the development of more effective targeted therapies. Out of these, clinical information was available for 155 patients (**Supplementary Table S1**). TARGET’s multi-omic framework offers a detailed molecular profile of childhood cancers. In addition, we assembled clinical data and assessed the expression of 84 genes previously implicated in cuproptosis.

### 2.2 Consensus clustering and survival analysis

Using the “ConsensusClusterPlus” R package, we categorized samples by analyzing the expression levels of 84 CRGs and their associated hazard ratios, detailed in **Supplementary Table S2** (10). The clustering parameter maxK was set to 5, and the optimal clustering method was determined by comparing consistency heatmaps across different clustering approaches. Following this classification, we utilized principal component analysis (PCA) to evaluate the clustering patterns and ensure the effectiveness of the grouping. To gain deeper insight into the clinical implications of these clusters, the “survival” package in R was used to examine their prognostic relevance. Kaplan-Meier survival analysis was subsequently performed to compare the survival differences between the identified CRGs clusters.

### 2.3 Enrichment analysis of genes: KEGG and GO

To gain a deeper understanding of the functional roles and associated pathways of the screened genes, we utilized the R package “clusterProfiler” for a comprehensive analysis. This tool was applied to investigate both Gene Ontology (GO) terms and Kyoto Encyclopedia of Genes and Genomes (KEGG) pathways, offering valuable insights into the biological processes, molecular functions, and cellular components linked to the identified genes. In the analysis, a p-value of less than 0.05 was considered significant. The results shed light on the functional annotations and pathways that are critical to understanding the underlying mechanisms influenced by these genes.

### 2.4 Evaluation of prognostic model

In order to estimate the risk ratio, we performed Cox proportional hazards regression by combining the differentially expressed gene data from cAML patients with their corresponding survival data. The patients were then randomly assigned into two groups: a training cohort consisting of 77 patients and a testing cohort of 78 patients. To identify potential risk factors within the training group, we first applied univariate Cox regression (p < 0.05) to examine the expression of CRGs. The LASSO Cox regression method was then applied to reduce overfitting and select key genes for the model. Following the multivariate Cox regression, we determined the coefficients for the prognostic model and assessed the relevance of each clinical feature in predicting patient outcomes. Based on the median risk score, the training group was classified into low-risk and high-risk categories. The performance of the prognostic model was assessed through Kaplan-Meier analysis, ROC curve analysis, risk score distribution, and survival status evaluation.

### 2.5 Analysis of drug sensitivity and correlations

Using the R package “pRRophetic,” we conducted Spearman’s rank correlation test to examine the relationship between risk scores and drug sensitivity. Spearman’s rank correlation was chosen due to its suitability for assessing monotonic relationships between variables, especially in cases where the data may not follow a normal distribution. Additionally, the package predicted IC50 values based on cancer cell line data, providing crucial drug sensitivity information (11).

### 2.6 Immune infiltration analysis

To assess the variety of immune cell types and functions present in the tumor microenvironment, we applied single-sample gene set enrichment analysis (ssGSEA). This approach utilizes 547 biomarkers to differentiate 29 distinct immune cell phenotypes (types and functions), such as T-cells, MHC class I, T cell co-stimulation, B-cells, type I/II IFN responses, plasma cells, and various subpopulations (12). The method relies on support vector regression and deconvolution techniques to analyze immune cell subtype expression data. By employing ssGSEA, we estimated the relative proportions of these immune-infiltrating cells in patient samples.

To ensure greater accuracy and to cross-validate the results obtained from ssGSEA, we subsequently used the CIBERSORT algorithm. This tool was employed to determine the composition of immune cells in the tissue samples, with analysis limited to those with p-values below 0.05 (13). In addition to these steps, we performed correlation analyses to explore how gene expression levels were associated with the proportions of different immune cell types.

### 2.7 Cell culture and *CNN3* knockdown

THP-1 and MOLM13 cells were cultured in RPMI-1640 medium (1640; Sigma-Aldrich, China; Merck KGaA, China), supplemented with 10% fetal bovine serum (FBS; Gibco, China; Thermo Fisher Scientific Inc., China) and 1% penicillin-streptomycin (Gibco). For the knockdown of *CNN3* expression, a short-hairpin RNA (shRNA) sequence was designed and employed. The shRNA sequence targeting CNN3 was as follows: Forward: CCGG GATTACCAATATAHCHACCAA CTCGAG TTGGTCGCTATATTGGTAATC TTTTTG; Reverse: AATT CAAAAA GATTACCAATATAGCGACCAA CTCGAG TTGGTCGCTATATTGGTAATC. Additionally, the scrambled shRNA sequence was referred to as plasmid No. 1864. Lentiviral transduction was used to achieve *CNN3* knockdown in the cells, utilizing Plasmid No. 12260 (psPAX2), Plasmid No. 12259 (pMD2.G), and Plasmid No. 10879 (pLKO.1-TRC control). The transduction process involved centrifugation at 1,000 rpm for 1.5 hours at 37°C with a polybrene solution at 8 µg/ml (Sigma-Aldrich; Merck KGaA). Afterward, the cells were subjected to puromycin selection (1 µg/ml; ST551, Beyotime, China) for 48 hours to ensure successful transduction. Finally, qRT-PCR was employed to validate the knockdown efficiency of CNN3.

### 2.8 qRT-PCR experiment

Cells were sorted into 1 ml of Trizol reagent (Thermo Fisher Scientific, 15596026) for RNA extraction and quantified using a NanoDrop ND-100 spectrophotometer (Thermo Fisher Scientific). cDNA was synthesized using the HiScript II one-step qRT-PCR Kit (Vazyme, Q221-01) on a CFX96 real-time PCR system (Bio-Rad), with β-actin as the internal control. The primer sequences for *CNN3* were: Forward: GAAGAAGGTCAACGAGTCCTCA; Reverse: AGTCTGAACCTGGGTCATGTT.

### 2.9 Flow cytometry

In order to evaluate cell apoptosis, we stained THP-1 (human monocytic leukemia cells) and MOLM13 (human acute myeloid leukemia cells) cells using Annexin V. For measuring intracellular ROS levels, the same cell lines were stained with H2-DCFDA (D6883, Sigma) and subsequently analyzed using fluorescence-activated cell sorting (FACS). Cells transfected with the vehicle were used as controls to ensure proper gating and data interpretation. The cell sorting and analysis were carried out with an Attune NxT analyzer (Thermo Fisher Scientific). The resulting data from these procedures were then processed and analyzed with FlowJo software.

## 3 Results

### 3.1 cAML consensus clustering and identification of differentially expressed genes using CRGs

**Figure 1:**
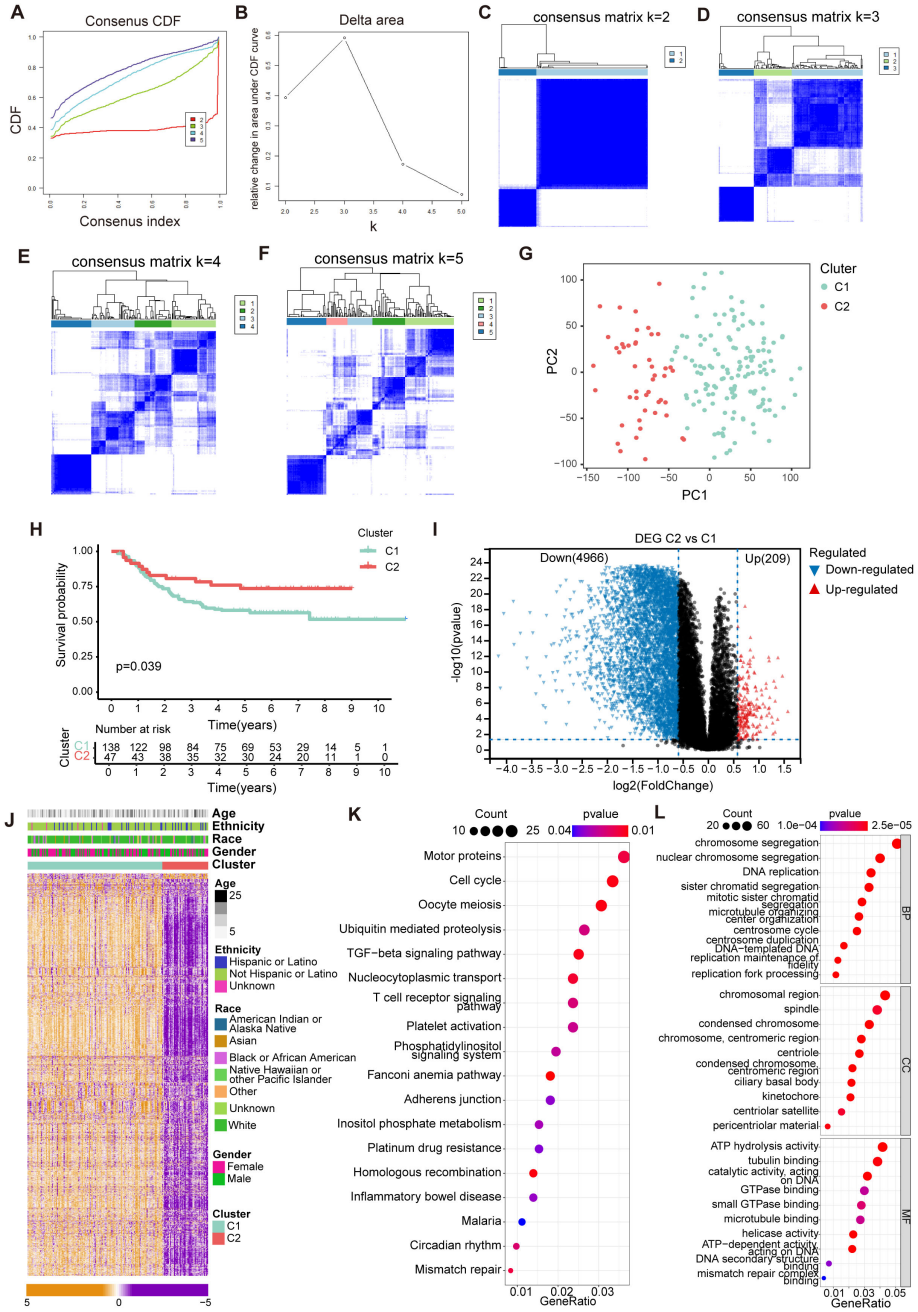
Identification of cuproptosis-related molecular subtypes and comprehensive pathway enrichment analysis in cAML. **(A)** CDF curves displayed consensus distributions from k=2 to k=5. **(B)** Area fraction under the CDF curve for k =2–5. The horizontal axis indicated the number of categories (k), while the vertical axis indicated the relative changes in the area under the CDF curves. **(C-F)** Consensus clustering matrixes were generated for values of k ranging from 2 to 5. **(G)** The PCA plot showing the distribution between groups divided by cuproptosis-related genes. **(H)** KM cures in two groups separated by cuproptosis-related genes. **(I)** Volcano plot showing up/down-regulated DEGs of the Cluster 2 vs. Cluster 1. Blue dots represent down-regulated DEGs while red dots represent up-regulated DEGs. **(J)** Heatmap showing the distribution of DEGs of cuproptosis-related genes in cAML. **(K, L)** Bubble chart showing KEGG and GO enrichment for DEGs of Cluster 1 vs. Cluster 2.

In our investigation, we focused on 84 CRGs identified from previous studies to evaluate their prognostic significance in cAML using data from the TARGET database. We categorized the samples based on CRGs expression levels, which allowed us to employ consensus clustering and successfully identify two distinct clusters at k=2 (**Figures 1A–F**). This clustering analysis was crucial for distinguishing between different gene expression profiles. The differentially expressed genes (DEGs) between these clusters were further validated, providing a clearer classification framework based on CRGs expression (**Figure 1G**). The Kaplan-Meier survival analysis highlighted significant survival differences between the two clusters (p < 0.05), underscoring the clinical relevance of these gene groups (**Figure 1H**).

To visually represent the DEGs distribution, we utilized a volcano plot, which revealed that Cluster 2 had 209 upregulated genes, and 4966 downregulated genes compared to Cluster 1 (**Figure 1I**). Complementing this, a heatmap displayed the distribution of DEGs across the clusters (**Figure 1J**). Functional enrichment analyses, including KEGG and GO, pointed out that the DEGs were predominantly involved in key biological processes such as immune regulation, cell cycle progression, and chromosome activation (**Figures 1K, L**). These findings suggest that the identified CRGs play significant roles in cAML pathogenesis by influencing critical cellular functions and immune responses.

### 3.2 Assessment of prognostic model using cuproptosis-related genes in cAML patients

**Figure 2:**
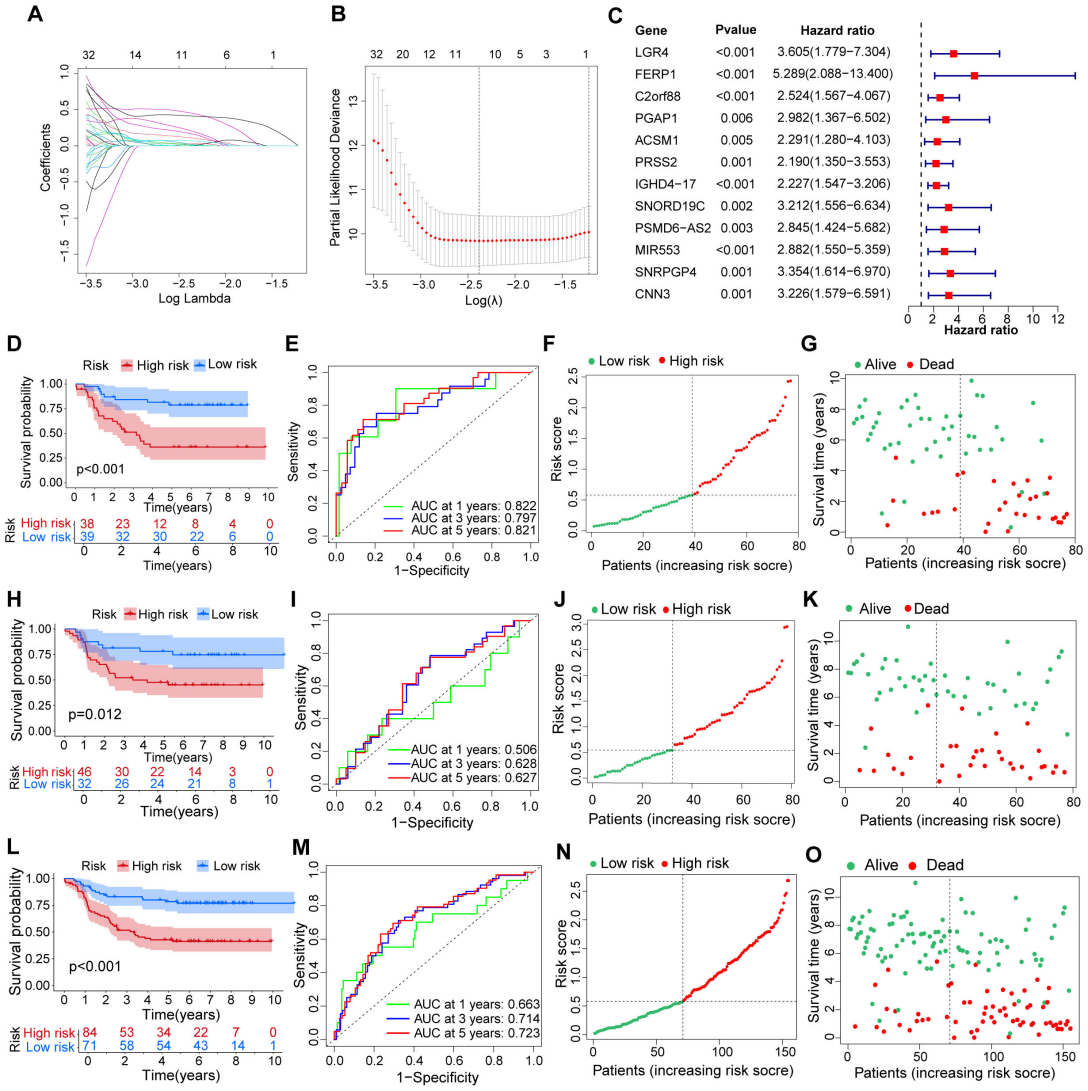
Evaluation for a prognostic model of genes related to cuproptosis in patients with cAML. **(A)** Plots of LASSO selecting candidate genes. **(B)** Cross-validation for LASSO. **(C)** The forest plot displays the hazard ratios of risk genes in the prognostic model for cAML patients. **(D–G)** The figures comprise ROC, KM curve, plots showing distribution between risk score and patients, and plots illustrating distribution between survival status and patients in the training group. **(H-O)** The types of **(H-O)** are consistent with **(D–G)**, but the group is a testing group and a merging group, respectively.

Through the use of LASSO regression and random forest algorithms, we identified 12 critical genes linked to cAML (**Figures 2A–C**). These genes—*LGR4*, *FERP1*, *C2orf88*, *PGAP1*, *ACSM1*, *PRSS2*, *IGHD4-17*, *SNORD19C*, *PSMD6-AS2*, *MIR553*, *SNRPGP4* and *CNN3*—were further validated as the most significant predictors of cAML (p < 0.01) prognosis through Cox and LASSO Cox regression analysis. The formula for the prognostic model is detailed in **Supplementary Table S3**. To ensure the reliability and accuracy of the model, it was thoroughly tested across different patient groups, including the training, testing, and merged cohorts. As expected, significant survival differences were observed, with p-values less than 0.001 for the training and merged groups, and p < 0.05 for the testing group.

The robustness of the model was further confirmed by analyzing the ROC curve AUCs, which provided a detailed assessment of the model’s predictive performance. For the training group, the AUCs were 0.822 at 1 year, 0.797 at 3 years, and 0.821 at 5 years, indicating strong predictive accuracy. The testing group showed lower AUCs of 0.506, 0.628, and 0.627, respectively, while the merged group exhibited moderate performance with AUCs of 0.663, 0.714, and 0.723 (**Figures 2D–O**). These results underscore the model’s ability to distinguish between high- and low-risk patients, with high-risk individuals demonstrating significantly poorer survival outcomes compared to those in the low-risk group.

This in-depth analysis underscores the model’s clinical relevance in categorizing cAML patients according to their risk profiles, providing key insights for developing personalized treatment strategies.

### 3.3 Analysis of drug sensitivity and risk score

**Figure 3:**
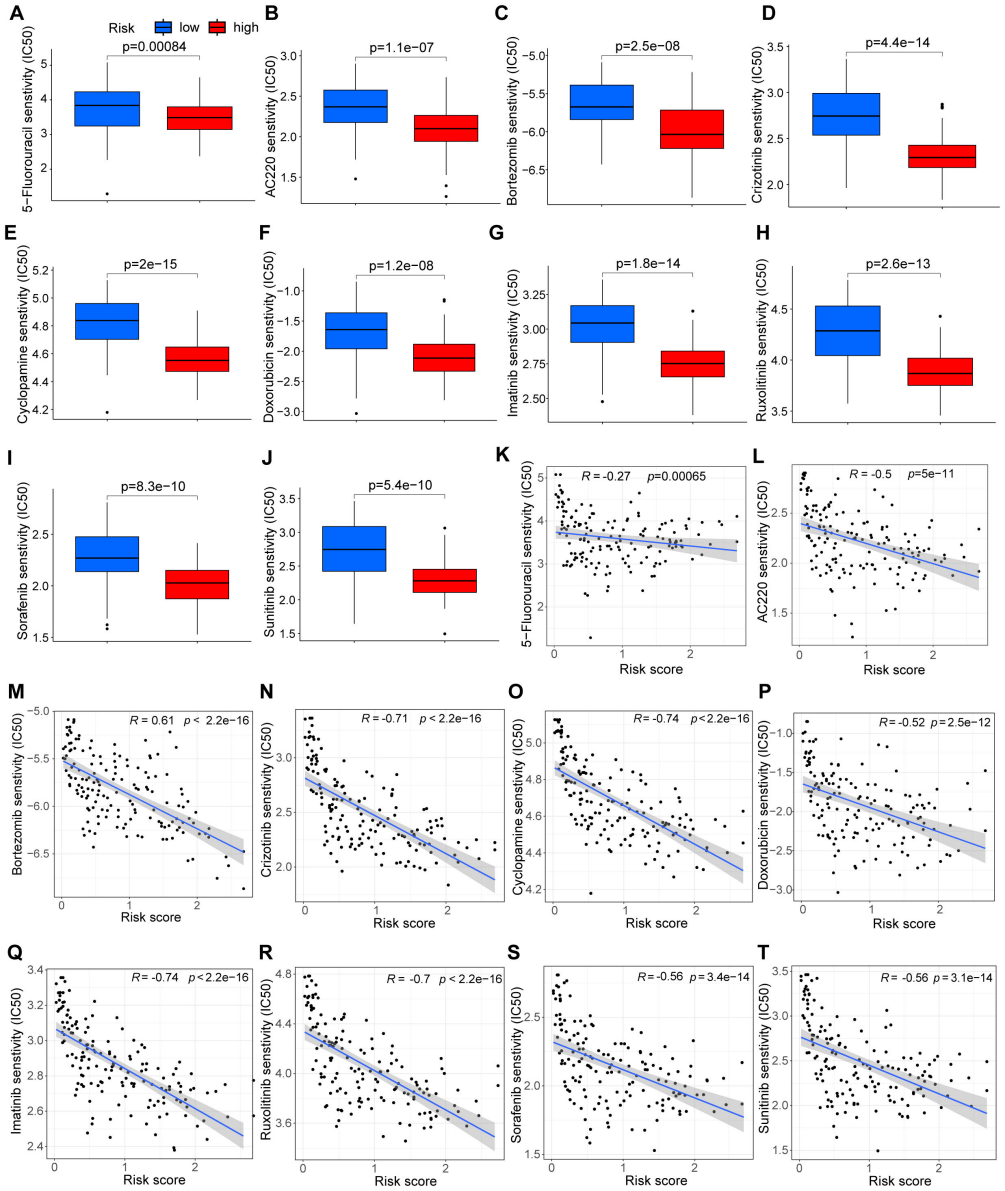
Analysis of drug sensitivity and risk score. **(A–J)** Drug sensitivity between groups divided by risks. **(K–T)** Correlation between drug sensitivity and risk score.

Due to the challenge of chemotherapy resistance in relapsed cAML, we evaluated the effectiveness of commonly used leukemia treatments to determine their potential sensitivity. Based on the study’s findings, high-risk patients are predicted to be sensitive to drugs such as 5-Fluorouracil, quizartinib (AC220), and bortezomib, suggesting their potential effectiveness in relapsed cAML cases (**Figures 3A–J**). Correlation analysis further showed that higher risk scores were linked to lower IC50 values for these drugs, suggesting their potential efficacy in relapsed cAML cases (**Figures 3K–T**).

### 3.4 Characteristics of immune infiltration in the risk model

**Figure 4:**
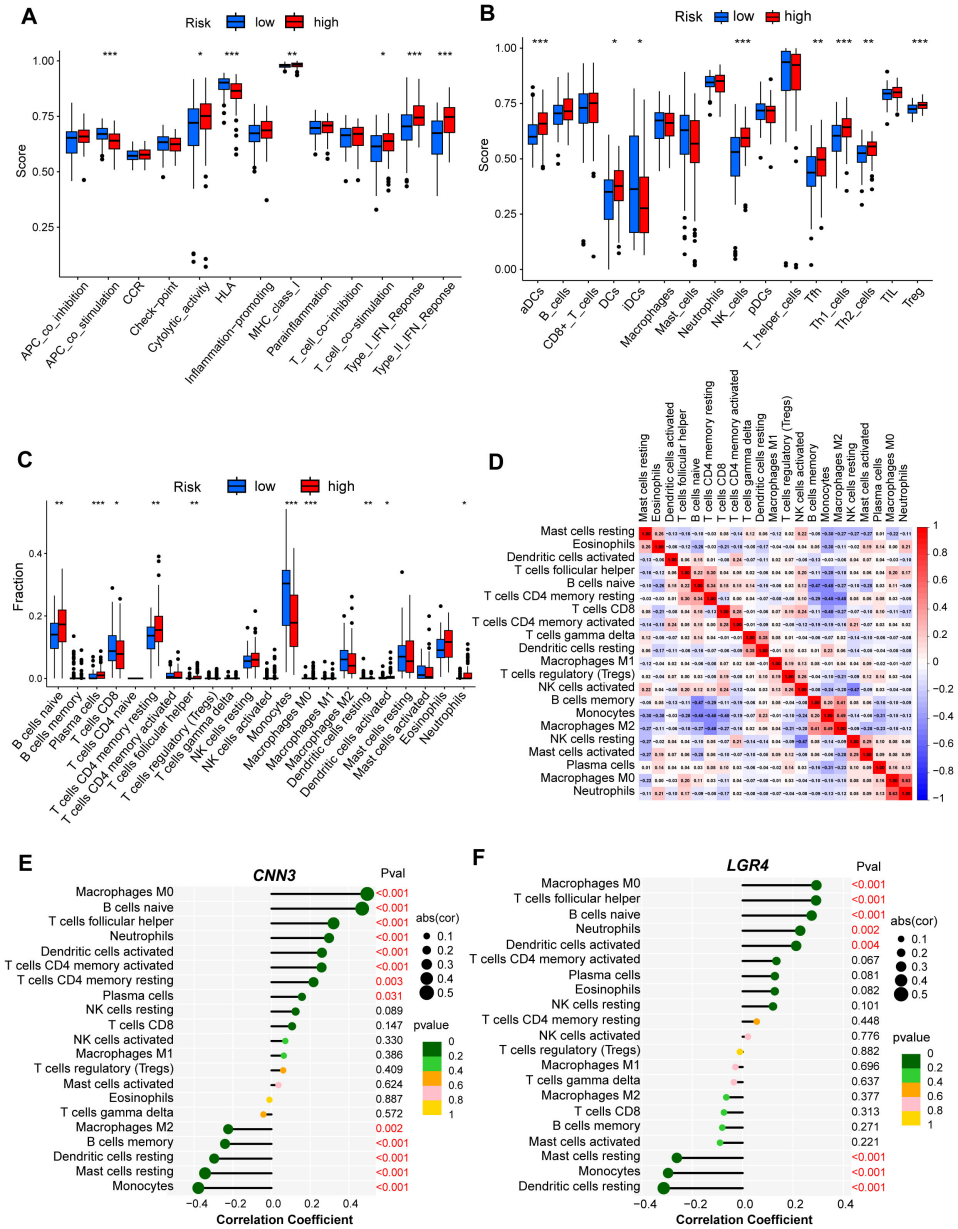
Analysis of immune infiltration for hub genes from low- and high-risk group of cAML. **(A, B)** Box plot showed the difference of immune infiltration assessed with ssGSEA in low-risk compared to high-risk group. **(C)** Box plot showed the difference of immune cell subtypes validated by CIBERSORT algorithm in low-risk compared to high-risk group. P-values in **(A-C)** are denoted by asterisks: *P<0.05, **P<0.01, ***P<0.001. **(D)** Correlation analysis heatmap between various types of immune cells. **(E, F)** Bubble map for the correlations between hub genes from two CCMs clusters and immune cells. Circles on the right indicates the absolute value of the correlation coefficient. The bigger the circle, the stronger the positive/negative correlation. P-value was indicated by color. The deeper of the green color, the closer the P-value was to zero.

The immune microenvironment, composed of various elements such as immune cells, extracellular matrix, growth factors, and inflammatory mediators, plays a pivotal role in the development and recurrence of cAML. To investigate the underlying molecular mechanisms, we conducted an ssGSEA analysis to explore the relationship between immune infiltration and the two risk groups. Our findings revealed significant variations in several immune functions (p < 0.05), including APC co-stimulation, cytolytic activity, HLA, MHC class I, T cell co-stimulation, and type I/II IFN responses between the low- and high-risk groups (**Figure 4A**). These functional differences point to the diverse ways in which the immune microenvironment influences disease progression.

Additionally, we examined the types of immune cells present in both risk groups and found higher levels of immune infiltration in the high-risk group, particularly in aDCs, DCs, iDCs, NK cells, Tfh cells, Th1, Th2, and Treg cells (**Figure 4B**). This heightened infiltration suggests that the immune system’s role in cAML is more prominent in patients with higher risk scores. To ensure the robustness of these results, we employed the CIBERSORT algorithm, which confirmed the consistency of immune cell composition patterns observed in the ssGSEA analysis (**Figure 4C**).

Furthermore, to understand potential interactions within the immune microenvironment, we performed correlation analysis among the immune cells, offering insights into how different cell types might collaborate or oppose one another in shaping the disease landscape (**Figure 4D**). Of particular interest, CNN3 and LGR4 genes were analysed in relation to immune cell infiltration, and both exhibited strong associations with distinct immune cell populations. These results underscore the critical roles of CNN3 and LGR4 in modulating immune responses and suggest that they are integral to the immune landscape of cAML (**Figures 4E, F**).

### 3.5 Verification of the *CNN3* gene’s role in cell line models

**Figure 5:**
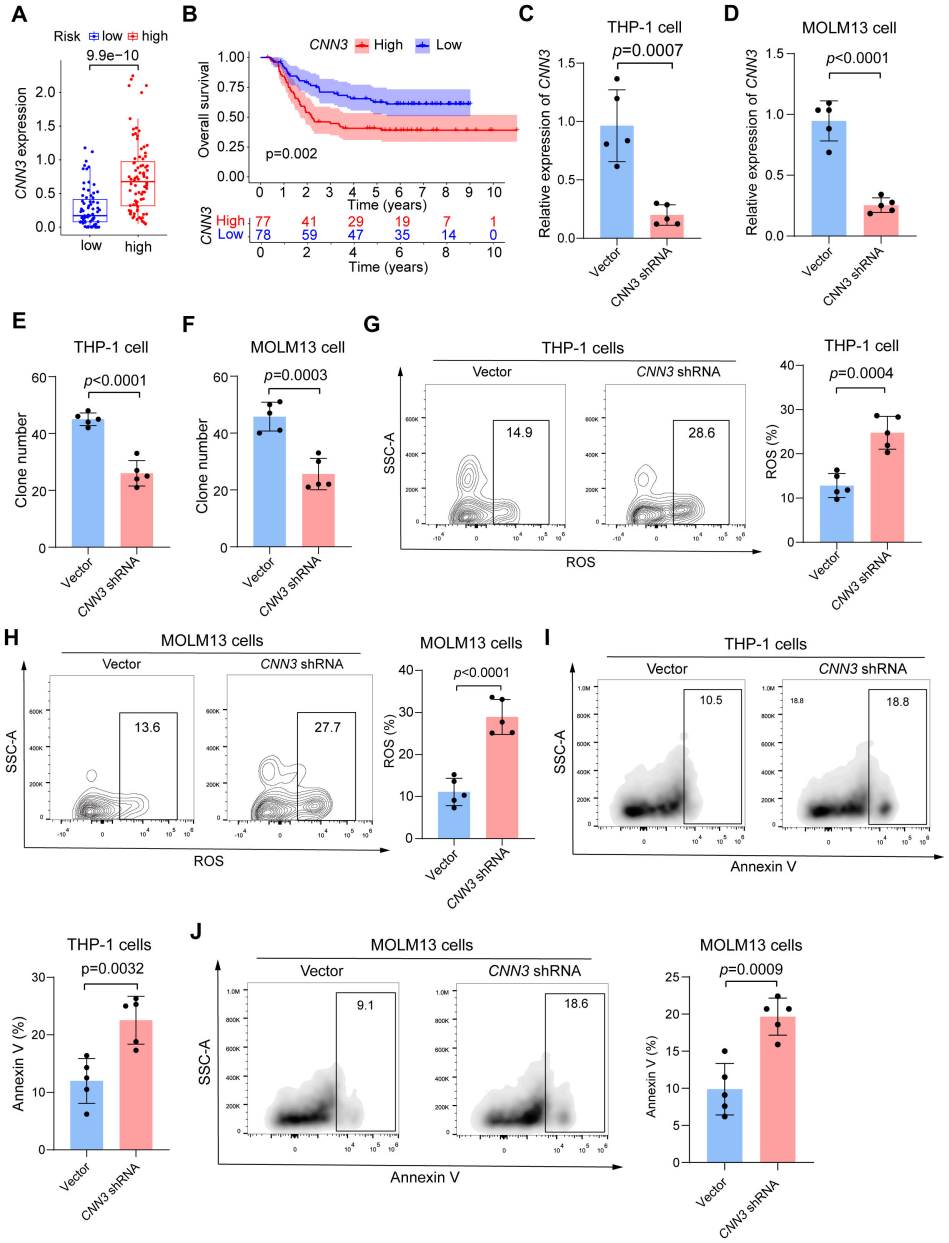
**(A)** Box plot of the expression level of CNN3 in low- and high-risk groups in cAML patients. **(B)** Kaplan-Meier curves for groups divided by median expression levels of CNN3. **(C, D)** Results of qPCR for CNN3 knockdown THP-1 and MOLM13 cells. **(E, F)** Colony-forming units for CNN3 knockdown THP-1 and MOLM13 cells. **(G, H)** Analysis of ROS level for CNN3 knockdown THP-1 and MOLM13 cells. **(I, J)** Analysis of apoptosis for CNN3 knockdown THP-1 and MOLM13 cells.

Our preliminary analysis showed that *CNN3* was highly expressed in the high-risk group, and this variation in expression contributed to the observed differences in survival outcomes between the groups (**Figures 5A, B**). Given *CNN3*’s significant involvement in immune-related processes and its critical role in our prognostic model (p < 0.001), we carried out a series of experiments to explore its function in cAML. First, shRNA was designed to knock down *CNN3* in THP-1 and MOLM13 cell lines, with successful knockdown confirmed via qRT-PCR (**Figures 5C, D**). Colony-forming unit (CFU) assays showed a substantial reduction in leukemia cell proliferation following *CNN3* knockdown compared to controls (**Figures 5E, F**). Additionally, flow cytometry was employed to measure reactive oxygen species (ROS) levels and apoptosis, revealing that *CNN3* knockdown led to significantly increased ROS levels and apoptosis in both cell lines (p < 0.05) (**Figures 5G-J**).

## 4 Discussion

While copper-induced cell death has been widely studied across various cancers, its role in cAML remains unclear. Despite notable advances in cAML treatment, some patients still do not benefit from current therapies and experience poor prognoses (14). Investigating the potential role of cuproptosis in cAML could offer novel therapeutic insights.

Through a combination of bioinformatics analysis and experimental validation, we examined the impact of CRGs on cAML, particularly focusing on their involvement in immune response, drug resistance, and patient prognosis. Using sequencing data from the TCGA database, we constructed a prognostic model that categorized patients into two distinct subtypes, each demonstrating significant differences in survival outcomes. Further analysis of DEGs revealed their association with key processes such as immune regulation, cell cycle control, and chromosome activation, all of which are critical for cancer cell proliferation. These findings suggest a potential role for CRGs in driving abnormal hematopoietic cell proliferation through mechanisms like chromosome replication and cell cycle dysregulation, ultimately contributing to the development of leukemia.

We developed a prognostic risk model for the two subtypes using univariate, LASSO, and multivariate logistic regression, identifying 12 risk genes. The observed lower predictive performance of the prognostic model in the testing group, compared to the training and merged groups, may be attributed to the limited sample size of cAML patients and the heterogeneity between samples. We then examined drug resistance patterns in patients with poor prognosis. Interestingly, drug sensitivity predictions suggest that several leukemia treatments may remain effective in these patients. Our findings also highlight the role of CRGs in cAML resistance. Additionally, immune function and immune cells were analyzed based on risk groups, revealing immunomodulatory effects of *CNN3* and *LGR4*, with *LGR4* known to maintain AML stem cell function (15, 16). Further validation in AML cell lines confirmed *CNN3* as a key factor in the prognostic model, associated with poor prognosis.

Calponin 3 (CNN3), part of the CNN protein family, is defined by its tandem repeat sequences (17). *CNN3* has an acidic C-terminus and a highly homologous basic N-terminus with another family member, CNN1. This protein family is linked to the cytoskeleton but does not participate in contraction (18). In addition to remodeling actin stress fibers (19), members of the CNN family regulate gastric and ovarian cancers (20, 21). Recent studies suggest that *CNN3* acts as an oncogene in gastric, colorectal, and cervical cancers, and is associated with cancer cell drug resistance (22–24). Furthermore, it serves as a diagnostic marker for lymph node metastasis in colorectal cancer (25). Previous research has shown that knockdown of *CNN3* inhibits extracellular signal-regulated kinase 1/2 (ERK1/2) and p38 phosphorylation, reducing osteosarcoma cell activity and proliferation via the mitogen-activated protein kinase (MAPK) signaling pathway. Interestingly, although *CNN3* is generally considered an oncogene, research in non-small cell lung cancer suggests that *CNN3* expression suppresses cancer cell proliferation and metastasis (26), indicating that *CNN3*’s role in cancer progression may be context-dependent. Louka E et al. reported that *CNN3* expression is significantly higher in the blood of juvenile myelomonocytic leukemia patients compared to cord blood (27). However, the role of *CNN3* in cAML progression remains unclear and warrants further investigation. Our analysis and experimental results consistently suggest *CNN3*’s potential oncogenic role in cAML and its ability to provide survival advantages to cancer cells.

Cuproptosis, a copper-dependent form of programmed cell death, offers a novel approach in cancer therapy by diversifying cell death mechanisms, potentially reducing resistance and improving treatment outcomes (28). Numerous CRGs have been identified, with context-dependent roles in cancer, influencing neoplasm-immunity interactions and chemosensitivity (29–31). These genes act as risk or protective factors in patient outcomes and are involved in tumorigenesis pathways, emphasizing their clinical significance and potential as therapeutic targets (32, 33). Copper ionophores that induce cuproptosis, such as dimercaprol (DSF) and elesclomol (ES), have demonstrated safety in clinical trials, though their efficacy in unselected populations remains to be confirmed (34). The interplay between cuproptosis and other regulated cell death forms, like necroptosis and ferroptosis, and its impact on immunotherapy responses highlight cuproptosis as a multifaceted component of cancer management (35). AML features genetic mutations that lead to excessive proliferation and evasion of regulated cell death, with apoptosis and ferroptosis being key pathways. Apoptosis, regulated by the B-cell lymphoma 2 (BCL-2) proteins, is crucial in preventing tumorigenesis and drug resistance, making it a target for new therapies. Ferroptosis, driven by iron metabolism, oxidative stress, and lipid metabolism, is related to the survival and maintenance of AML cells (36). Further investigation into CRGs in the tumor microenvironment, their relationship with drug resistance, and their predictive value for chemotherapy response underscores the complexity of cuproptosis in cancer pathophysiology (37). Previous studies have found that high levels of copper are toxic to cells and can induce cuproptosis, potentially through the induction of oxidative stress by elevating ROS levels beyond a sustainable threshold within the cells (38). Flow cytometry detected excessive levels of ROS and Annexin V in AML cells, indicating the presence of intense oxidative stress within the cells and triggering cell death. Therefore, CRGs expression levels influence chemotherapy outcomes and reflect the prognosis of cAML patients. As an important mechanism in cancer progression, we identified *CNN3* as a key cuproptosis-related signature affecting cAML development. There may be shared regulatory pathways between cuproptosis and *CNN3*. Our study provides evidence supporting the association of *CNN3* downregulation with improved prognosis in cAML patients, highlighting its potential as a predictive marker.

A comparison of the CIBERSORT and ssGSEA analysis results revealed a higher level of Dendritic cells (DCs) activation in the high-risk group compared to the low-risk group. DCs, a specialized type of antigen-presenting cell, are crucial for initiating and regulating immune responses. The immune response to exogenous antigens can be triggered by the ability of DCs to capture, process, and deliver antigens from infected or tumor cells to T cells (39). Interestingly, despite the increased activation of DCs in the high-risk group, this was associated with poorer prognosis, which contrasts with findings in adult AML patients (40). This result strongly suggests that when considering immune-based therapies, such as DCs vaccines, in pediatric AML patients, the differences in immune responses between pediatric and adult patients should be carefully taken into account. Our analysis provides new insights into immunotherapy for cAML, highlighting the need to carefully evaluate the feasibility of conventional immunotherapies in the context of cAML.

This study has several limitations. The limited availability of publicly accessible cAML datasets, coupled with small sample sizes, may hinder the representativeness of the overall cAML patient population. Future studies with larger and more diverse cohorts are needed to validate our findings and enhance their generalizability. Clinically, these findings hold promise for advancing personalized treatment strategies. The incorporation of cuproptosis-related genes like *CNN3* into prognostic models could enable better risk stratification of cAML patients. Additionally, *CNN3*’s involvement in immune-related mechanisms and copper-dependent cell death pathways may provide novel therapeutic targets for high-risk patients. However, larger studies and *in vivo* validation are essential before clinical applications can be realized.

## 5 Conclusion

In summary, this study explored the role of CRGs in cAML and developed a prognostic model, identifying 12 associated risk genes. Our analysis and experimental findings highlight *CNN3* as a key factor in cAML prognosis, with its knockdown improving outcomes in AML models, suggesting *CNN3* as a potential therapeutic target for cAML.

## Data Availability

Publicly available datasets were analyzed in this study. This data can be found here: https://portal.gdc.cancer.gov/.
